# Bola3 Regulates Beige Adipocyte Thermogenesis *via* Maintaining Mitochondrial Homeostasis and Lipolysis

**DOI:** 10.3389/fendo.2020.592154

**Published:** 2021-01-11

**Authors:** Ningning Bai, Jingyuan Ma, Miriayi Alimujiang, Jun Xu, Fan Hu, Yuejie Xu, Qingyang Leng, Shuqing Chen, Xiaohua Li, Junfeng Han, Weiping Jia, Yuqian Bao, Ying Yang

**Affiliations:** ^1^Department of Endocrinology and Metabolism, Shanghai Clinical Center for Diabetes, Shanghai Key Clinical Center for Metabolic Disease, Shanghai Diabetes Institute, Shanghai Key Laboratory of Diabetes Mellitus, Shanghai Jiao Tong University Affiliated Sixth People’s Hospital, Shanghai, China; ^2^Department of Geriatrics, Shanghai Jiao Tong University Affiliated Sixth People’s Hospital, Shanghai, China; ^3^Department of Endocrinology, Seventh People’s Hospital of Shanghai University of TCM, Shanghai, China

**Keywords:** BolA family member 3, mitochondria, beige adipocyte, lipolysis, thermogenesis

## Abstract

Mitochondrial iron-sulfur (Fe-S) cluster is an important cofactor for the maturation of Fe-S proteins, which are ubiquitously involved in energy metabolism; however, factors facilitating this process in beige fat have not been established. Here, we identified BolA family member 3 (Bola3), as one of 17 mitochondrial Fe-S cluster assembly genes, was the most significant induced gene in the browning program of white adipose tissue. Using lentiviral-delivered shRNA *in vitro*, we determined that Bola3 deficiency inhibited thermogenesis activity without affecting lipogenesis in differentiated beige adipocytes. The inhibition effect of Bola3 knockdown might be through impairing mitochondrial homeostasis and lipolysis. This was evidenced by the decreased expression of mitochondria related genes and respiratory chain complexes, attenuated mitochondrial formation, reduced mitochondrial maximal respiration and inhibited isoproterenol-stimulated lipolysis. Furthermore, BOLA3 mRNA levels were higher in human deep neck brown fat than in the paired subcutaneous white fat, and were positively correlated with thermogenesis related genes (UCP1, CIDEA, PRDM16, PPARG, COX7A1, and LIPE) expression in human omental adipose depots. This study demonstrates that Bola3 is associated with adipose tissue oxidative capacity both in mice and human, and it plays an indispensable role in beige adipocyte thermogenesis *via* maintaining mitochondrial homeostasis and adrenergic signaling-induced lipolysis.

## Introduction

Obesity occurs when energy intake chronically exceeds energy expenditure, which increases the risk of developing metabolic disorders, such as insulin resistance, type 2 diabetes and cardiovascular diseases (1, 2). Adipose tissues play a pivotal role in systematic energy homeostasis through multiple functions (3). There are three functionally distinct adipocyte subtypes that have been identified in mice. Classically, white adipocytes are responsible for triglyceride storage and mobilization as needed; brown adipocytes contain abundant uncoupling protein 1 (UCP1), which uncouples fuels oxidation from ATP production to heat generation; beige adipocytes are interspersed within white adipose tissue (WAT) and express UCP1 upon thermogenic activation (4). Although brown and beige adipocytes share many multilocular lipid droplets and densely packed mitochondria, they also have distinguishing developmental features. Unlike brown adipocytes, beige adipocytes originate from heterogeneous populations of adipogenic precursors with additional inducers (5). Thus, beige adipocytes have a flexible phenotype and can potentially carry out lipid turnover, either lipid storage or dissipation, depending on certain stimuli. Novel underlying mechanisms that promote beige adipocyte development need to be further defined to improve therapeutic avenues.

Beige adipocytes are highly inducible under both internal and external cues, including chronic cold exposure, PPARγ agonist, β**_3_**-adrenergic agonists, and exercise training, resulting in an improvement of insulin sensitivity (6). Although the underlying molecular mechanisms are not fully understood, it is widely acknowledged that mitochondrial biogenesis is the key to beige adipocyte development (7). Mitochondria play a central role in adipocyte metabolism by switching fuels toward fat storage or β-oxidation, and the latter can drive heat production in the presence of UCP1 (8). Mitochondrial dynamics are regulated by a balance between mitochondrial biogenesis and degradation, and dysregulation of mitochondrial biogenesis in adipose tissue is associated with obesity and diabetes, which have been known to exhibit decreased activities of electron transport chain and oxidative enzymes (9, 10). Conversely, specific improvement of mitochondrial function in adipocytes ameliorates metabolic dysfunction. For instance, fat-specific overexpression of mitoNEET, an outer mitochondrial membrane protein that contains iron-sulfur (Fe-S) clusters, ameliorates obesity-associated adipose dysfunction and exhibits a browning signature in inguinal WAT (iWAT), and thereby mice display a healthy phenotype (11, 12). Due to containing Fe-S clusters, mitoNEET has been identified as a Fe-S protein (13). The mitochondrial Fe-S cluster is an important cofactor for Fe-S proteins, which are ubiquitously involved in a wide range of cellular processes, including mitochondrial electron transport, enzymatic catalysis and regulation (14). These observations highlight a tight connection between mitochondrial Fe-S proteins and adipose function, while factors that facilitate the synthesis of Fe-S clusters and the maturation of Fe-S proteins in beige fat have not been fully established.

Mitochondria are the primary site for the synthesis of Fe-S clusters and the maturation of Fe-S proteins, which are assisted by 17 proteins forming the Fe-S cluster assembly machinery (15). These 17 proteins are encoded by nuclear genes and evolutionarily conserved from bacteria to human (16). As one of them, BolA family member 3 (Bola3) encodes a mitochondrial protein that facilitates Fe-S clusters insertion into a subset of mitochondrial Fe-S proteins, such as enzymes involved in metabolism and energy production (17). Mutation in human BOLA3 causes multiple mitochondrial dysfunctions syndrome, accompanied by defects in 2-oxoacid dehydrogenases and mitochondrial respiratory chain complexes (18, 19). The BolA family contains two other members: Bola1, like Bola3, is located to mitochondria and performs overlapping roles during the maturation of specific Fe-S proteins; while Bola2 is present in the cytosol and its yeast homolog Bol2 regulates iron metabolism (17, 20). The potential molecular function of BolA proteins in functional cell types, particularly Bola3, remains to be resolved. Recently, Bola3 was reported to act a connective role between Fe-S-dependent oxidative respiration and glycine homeostasis in the process of endothelial metabolic re-programming, which is critical for pulmonary hypertension pathogenesis (21). Beige adipocyte is a re-programmed type of white adipocyte upon certain stimuli, and it largely depends on mitochondrial oxidative phosphorylation to drive thermogenesis (22). However, the potential role for Bola3 in beige adipocyte development remains undefined yet.

In the present study, we aimed to elucidate the role and possible mechanism of Bola3 in beige adipocyte by *in vitro* loss of function experiments. The association analysis between BOLA3 levels and thermogenic gene program in human adipose depots were performed. We found that Bola3 expression was significantly induced in iWAT of mice upon thermogenic activation, and Bola3 deficiency impaired the thermogenesis capacity in beige adipocytes. Furthermore, we demonstrated that BOLA3 mRNA levels were positively correlated with the expression of thermogenic genes in human omental adipose tissue samples. Thus, these findings may provide more extensive information on the regulatory role for Bola3 in beige adipocyte development.

## Materials and Methods

### Data Processing

Transcriptome profiles of GSE86338 by high-throughput RNA sequencing (RNA-seq) were downloaded from Gene Expression Omnibus (GEO, https://www.ncbi.nlm.nih.gov/geo/). This dataset describes the transcriptional change during WAT browning induced by chronic cold exposure, β**_3_**-adrenergic agonist and intensive exercise, and brown adipose tissue (BAT) activation by acute cold exposure (4 °C for 6 h) and inactivation by thermoneutrality (TN, 30 °C for 7 days) in C57BL/6J mice (23). The gene-expression change (log**_2_**FC) was identified by EBSeq algorithms. Genes cluster involved in the process of iWAT browning and BAT activation/inactivation were listed in the form of heatmap.

Another transcriptome data by microarray were also downloaded from GEO, and the accession number is GSE10246 (24). This dataset contains gene expression data from diverse tissues from mice. The gene-expression difference was represented as log**_2_**FC, where FC was expressed as fold over the median value in the selected metabolic tissues. The tissue-specific expression profiles of genes cluster were summarized in a heatmap form.

The transcriptome analysis of the omental adipose samples from lean and obese human subjects were described in our previous study (25). In brief, sequencing libraries were first constructed and then RNA-seq was performed with the Illumina instrument at Shanghai Biotechnology Corporation. After sequences were mapped to the human genome (hg19), the reads were converted to fragments per kilobase of exon per million mapped reads (FPKM), which were calculated as mRNA level of each gene.

### Mice

Male C57BL/6 mice (SLAC, China) were bred on a chow diet with a 12-h light/dark cycle. For chronic cold exposure, 8-week-old mice were exposed to 4°C for 7 days; for β**_3_**-adrenergic agonist treatment, CL-316243(Sigma, C5976, 1 mg/kg) were implanted subcutaneously with mini-pumps for 7 days; mouse swimming exercise was implemented according to a previously established program (23). Briefly, the protocol started at 10 mins **two** times daily, with 10 mins increase each day until 90 mins, two times per day was reached. After that, the training lasted for 2 weeks. The water temperature was kept at 30 °C to avoid the effect of cold exposure. For iWAT denervation, surgery was performed on 3-month-old mice. An abdominal midline incision was made to expose iWAT, and then 10 µl of 6-hydroxydopamine (Sigma, H4381, 10 µg/µl freshly dissolved in saline) was administrated along one side of fat pad, and the other side was injected with saline, after which the skin incision was sutured (26). Two weeks after denervation, the mice were utilized for 7-day cold exposure. All animal procedures were approved by the Animal Care Committee of Shanghai Jiao Tong University Affiliated Sixth People’s Hospital.

### Human Adipose Tissue Samples

Human deep neck fat and the paired subcutaneous fat samples were obtained from metabolically heathy patients who underwent thyroidectomy at Shanghai Jiao Tong University Affiliated Sixth People’s Hospital. All patients had normal range of thyroid-stimulating hormone values and details on the collection were guided by the previous establishment (27). Human omental adipose tissue biopsies were collected from 10 (four men and six women) severely obese but metabolically healthy subjects with a BMI between 34 and 56, who underwent bariatric surgery at Shanghai Jiao Tong University Affiliated Sixth People’s Hospital, and 11 (three men and eight women) lean controls with a BMI between 18.5 and 24.9, all without metabolic diseases, who underwent a laparoscopic cholecystectomy at Shanghai Seventh People’s Hospital, as described in our recent study (25). This study was approved by the Human Research Ethics Committee of Shanghai Jiao Tong University Affiliated Sixth People’s Hospital and Shanghai Seventh People’s Hospital. All participants have signed written informed consent before taking part in this study.

### Stromal Vascular Fraction (SVF) Isolation and Cell Culture

SVF cells were isolated from iWAT of 6-week-old male mice as previously described (28). For the differentiation of beige adipocyte, SVF were grown to confluence and induced to differentiate with 0.5 mM IBMX (Sigma, I7018), 1 µM rosiglitazone (Sigma, R2408), 1 nM T3 (Sigma, T2877), 1 µM dexamethasone (Sigma, D4902) and 5 µg/ml insulin (Lily, HI0240) for two days, and then maintained with rosiglitazone, T3 and insulin for another four days (29). In some experiments, the differentiated beige adipocytes were treated for 12h with 0.5 mM dibutyryl-cAMP (Sigma, D0627) or for 12h with 20 µM H-89 (Sigma, B1427) and 10 µM SB202190 (Sigma, S7067).

### Gene Knock-Down by shRNA Lentivirus

Lentiviral shRNA clones for mouse Bola3 and a scrambled control were obtained from Shanghai GeneChem Corporation. For lentivirus production, 293T cells were transfected with 10 µg vectors. After 48h of incubation, the supernatant was collected. The SVF cells were infected with the virus supernatant to knock down Bola3, and the multiplicity of infection was 50. The efficiency of lentivirus infection was determined by the number of GFP-positive cells at 72h.

### RNA Extraction and Real-Time Quantitative PCR (RT-qPCR)

Total RNA was isolated from cultured cells and tissues by the Trizol reagent (Invitrogen, 15596018). 1 µg of total RNA was then converted to cDNA using the PrimeScript RT reagent Kit (Takara, RR047B). RT-qPCR amplification was conducted using SYBR Premix Ex Taq (Takara, RR820A) with a LightCycler480 system (Roche, Germany). Quantitative expression of targeted genes was normalized to housekeeping gene 36B4 and calculated using the 2^−ΔΔCT^ method. The primer sequences are summarized in **Table 1**.

**Table 1:** Primer Sequences Used in RT-qPCR.

Gene	Forward	Reverse
Mouse Bola3	CTGCGGGGCCATGTATGAAA	CCTGATTGACCATCTGGTGCT
Mouse Ucp1	AGGCTTCCAGTACCATTAGGT	CTGAGTGAGGCAAAGCTGATTT
Mouse Fabp4	AAGGTGAAGAGCATCATAACCCT	TCACGCCTTTCATAACACATTCC
Mouse Cidec	ATGGACTACGCCATGAAGTCT	CGGTGCTAACACGACAGGG
Mouse Pparg	TCGCTGATGCACTGCCTATG	GAGAGGTCCACAGAGCTGATT
Mouse Plin1	CTGTGTGCAATGCCTATGAGA	CTGGAGGGTATTGAAGAGCCG
Mouse 36B4	AAGCGCGTCCTGGCATTGTCT	CCGCAGGGGCAGCAGTGGT
Mouse Adipoq	TGTTCCTCTTAATCCTGCCCA	CCAACCTGCACAAGTTCCCTT
Mouse Glut4	GTGACTGGAACACTGGTCCTA	CCAGCCACGTTGCATTGTAG
Mouse Ppargc1a	TATGGAGTGACATAGAGTGTGCT	CCACTTCAATCCACCCAGAAAG
Mouse Cidea	TGACATTCATGGGATTGCAGAC	CATGGTTTGAAACTCGAAAAGGG
Mouse Cox7a1	CAGCGTCATGGTCAGTCTGT	AGAAAACCGTGTGGCAGAGA
Mouse Clstn3b	CTCCGCAGGAACAGCAGCCC	AGGATAACCATAAGCACCAG
Mouse Adrb3	TCTCTGGCTTTGTGGTCGGA	GTTGGTTATGGTCTGTAGTCTCG
Mouse Mfn1	CCTACTGCTCCTTCTAACCCA	AGGGACGCCAATCCTGTGA
Mouse Mfn2	AGAACTGGACCCGGTTACCA	CACTTCGCTGATACCCCTGA
Mouse Nrf1	AGCACGGAGTGACCCAAAC	TGTACGTGGCTACATGGACCT
Mouse Nrf2	CTTTAGTCAGCGACAGAAGGAC	AGGCATCTTGTTTGGGAATGTG
Mouse Tfam	ATTCCGAAGTGTTTTTCCAGCA	TCTGAAAGTTTTGCATCTGGGT
Mouse Fis1	TGTCCAAGAGCACGCAATTTG	CCTCGCACATACTTTAGAGCCTT
Human UCP1	GTGTGCCCAACTGTGCAATG	CCAGGATCCAAGTCGCAAGA
Human PPARGC1A	CAAGCCAAACCAACAACTTTATCTCT	CACACTTAAGGTGCGTTCAATAGTC
Human FABP4	CCTTTAAAAATACTGAGATTTCCTTCA	GGACACCCCCATCTAAGGTT
Human BOLA3	CACTTCACCATCGGATGTTTGC	GCTGTAGCTCGTGGAAACTTTT
Human RPLP0	AGCCCAGAACACTGGTCTC	ACTCAGGATTTCAATGGTGCC

### Western Blot

Protein samples were extracted with RIPA lysis buffer supplemented with phosphatase inhibitor cocktail (Roche, 4906845001) and protease inhibitor cocktail (Roche, 04693132001) and then subjected to western blot analysis according to the standard protocol. Membranes were incubated overnight at 4 °C with the following antibodies: UCP1 (Abcam, ab10983), PPARγ (Cell Signaling Technology, 2443), Perilipin 1 (Cell Signaling Technology, 3470), C/EBPα (Cell Signaling Technology, 2295), total OXPHOS rodent WB cocktail (Abcam, ab110413), p HSL Ser563 (Cell Signaling Technology, 4139), HSL (Cell Signaling Technology, 4107), Tubulin (Sigma, T6199), and GAPDH (KANGCHEN, KC-5G4). The antibody against BOLA3 was custom-produced by GenScript. Subsequently the membranes were incubated with secondary antibodies for 1h at room temperature. Protein bands were visualized with ECL Western HRP Substrate (Millipore, WBKLS0500) using Image Quant LAS4000 Imaging Systems (GE Healthcare, USA).

### Oil Red O Staining

After induction to mature beige adipocytes for six days, the differentiated cells underwent Red Oil O staining. In brief, cells were washed once with PBS, fixed in 4% paraformaldehyde for 15 mins, and stained with a filtered Oil Red O working solution (Sigma, O1391) for 20 mins. Subsequently, the stained cells were washed with PBS before imaging with microscope (Nikon Corp, Japan).

### Mitochondrial DNA Content

Genomic DNA was isolated from cultured adipocytes by the Gentra Puregene Cell Kit following the manufacturer**’**s instructions (Qiagen, 158745). The ratio of mitochondrial DNA (mtDNA) to genomic DNA was measured by performing qPCR. The following primers were used: mt-RNR1: forward 5**’**-AGGAGCCTGTTCTATAATCGATAAA-3**’**; reverse 5**’**-GATGGCGGTATATAGGCTGAA-3**’**. Genomic RBM15: forward 5**’**-GGACACTTTTCTTGGGCAAC-3**’**; reverse 5**’**-AGTTTGGCCCTGTGAGACAT-3**’**.

### MitoTracker Staining

Adipocytes were stained with MitoTracker Red probes (Invitrogen, M7513) in DMEM containing 0.25% BSA at 37°C for 30 mins. Cells were gently washed twice with PBS. Images were obtained with a fluorescence microscope (Nikon Corp, Japan).

### Cellular Triglyceride and Lipolysis Measurement

Triglyceride content in differentiated adipocytes was determined by kit (Shanghai Kehua Bio-Engineering, China) following manual instructions. For lipolysis measurement, adipocytes were pretreated with 1 µM isoproterenol (ISO) in DMEM containing 0.25% BSA for 3h. Glycerol content in the supernatant was measured using the reagent according to the manufacturer**’**s protocols. Results were standardized to total cellular protein content.

### Cellular Metabolic Rates

SVF cells were seeded on XF-24-well culture microplate (Seahorse Bioscience) pre-coated with poly-L-lysine and differentiated them into beige adipocytes for four days. Subsequently, Oxygen consumption rate (OCR) was measured using the Mito stress kit (Agilent, 103015) in an XF24 analyzer (Seahorse Bioscience). After measuring basal OCR, 2 µM oligomycin was added to measure the uncoupled respiration. Then, 1 µM FCCP was added to detect maximal respiration. Lastly, 1 µM rotenone/1 µM antimycin was added to measure non-mitochondrial respiration. Lastly, the final OCR results were standardized to total protein content.

### Statistical Analysis

All results were presented as means ± SEM from at least three independent experiments. Statistical differences between groups were calculated by unpaired and paired two-side Student**’**s t tests. Linear regression analysis was performed to examine the correlation between genes expression. *p < 0.05, **p < 0.01, and ***p ***<***0.001 compared with control group were considered as a statistical significance.

## Results

### Bola3 Expression Was Induced in the Browning Program of iWAT in Mice

**Figure 1:**
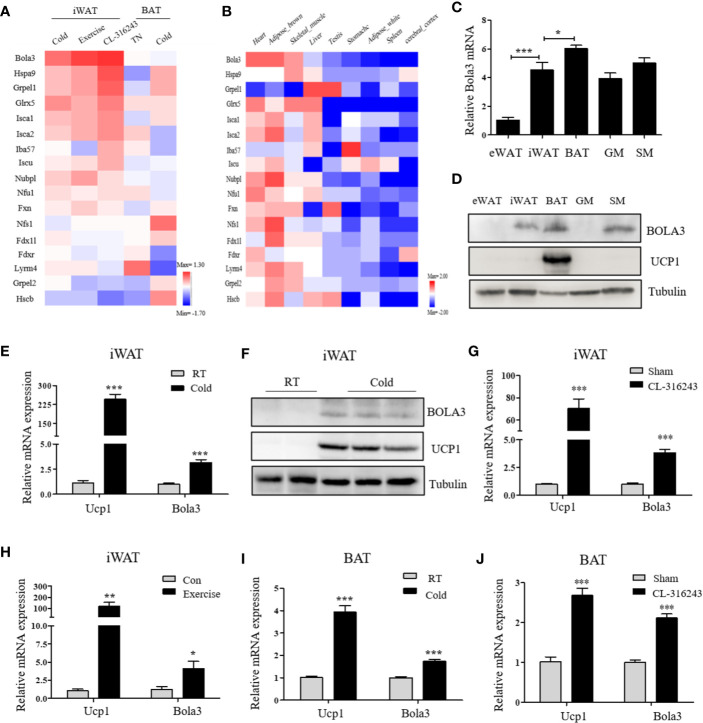
Bola3 expression was induced in inguinal WAT (iWAT) upon thermogenic activation. **(A)** Heatmap summarizing expression profiles (log**_2_**FC) of the mitochondrial Fe-S cluster assembly genes in the process of iWAT browning induced by cold exposure, exercise and CL-316243, and BAT activation/inactivation induced by acute cold and thermoneutrality (TN) respectively in C57BL/6J mice (GSE86338). **(B)** The expression patterns (log**_2_**FC) of Fe-S cluster assembly genes across major metabolic tissues in mice (GSE10246). **(C, D)** Metabolic tissues from 6-week-old male mice were assessed for Bola3 mRNA (n=4) and protein expression by qPCR and western blot. eWAT (epididymal WAT), iWAT (inguinal WAT), BAT (interscapular BAT), GM (Gastrocnemius muscle) and SM (Soleus muscle). **(E, F)** Relative mRNA (n=5) and protein levels of Bola3 and Ucp1 in iWAT from mice exposed to cold (4 °C) for 7 days in an individual cage, and mice housed at room temperature (RT) served as controls. **(G)** Relative mRNA expression of Bola3 and Ucp1 in iWAT from mice subjected to CL-316243 treatment or the sham operation (n=4). **(H)** Relative mRNA levels of Bola3 and Ucp1 in iWAT from exercise-trained and sedentary control mice (n=6). **(I)** Relative mRNA levels of Bola3 and Ucp1 in BAT under 7-day cold exposure (n=7). **(J)** Relative mRNA levels of Bola3 and Ucp1 in BAT under CL-316243 injection (n=4). Data were presented as means ± SEM. *p < 0.05, **p < 0.01, and ***p <0.001.

To identify the involvement of the mitochondrial Fe-S cluster assembly machinery in the browning program, we retrieved a transcriptome study (GSE86338) downloaded from GEO database and analyzed mRNA expression patterns of 17 Fe-S cluster assembly genes during WAT browning and BAT activation/inactivation in male C57BL/6J mice. Among these, Bola3 was the most significantly induced gene in iWAT under multiple thermogenic stimuli, while remained unchanged in acute regulation of BAT thermogenesis (**Figure 1A**). We also examined the mRNA profiles of these 17 genes across several mouse tissues by analyzing GSE10246, and found that most of them were highly expressed in mitochondria-enriched tissues, such as BAT and muscle tissues (**Figure 1B**). Then, the expression of Bola3 was validated in epididymal WAT (eWAT), iWAT, BAT, gastrocnemius muscle (GM), and soleus muscle (SM). We found that Bola3 mRNA and protein levels were most highly expressed in BAT, followed by SM and iWAT (**Figures 1C, D**). Furthermore, we confirmed that long-term cold exposure induced a significant induction of Bola3 transcript and protein levels in iWAT, which was similar to the effect of chronic cold exposure on Ucp1 expression (**Figures 1E, F**). Similarly, higher Bola3 mRNA expression was observed in iWAT from CL-316243-injected mice and exercise-trained mice compared to the controls, which were in consistent with Ucp1 expression (**Figures 1G, H**). Furthermore, we also found that Bola3 was transcriptionally upregulated in BAT under chronic cold exposure and CL-316243 injection, which had some discrepancies with acute cold treatment (**Figures 1I, J**). As the patterns of Bola3 expression could indicate important clues about its function, we hypothesized that Bola3 might play a specific role in beige fat development.

### Bola3 Expression in Beige Adipocytes Was Enhanced *via* Adrenergic Signaling Stimulation

**Figure 2:**
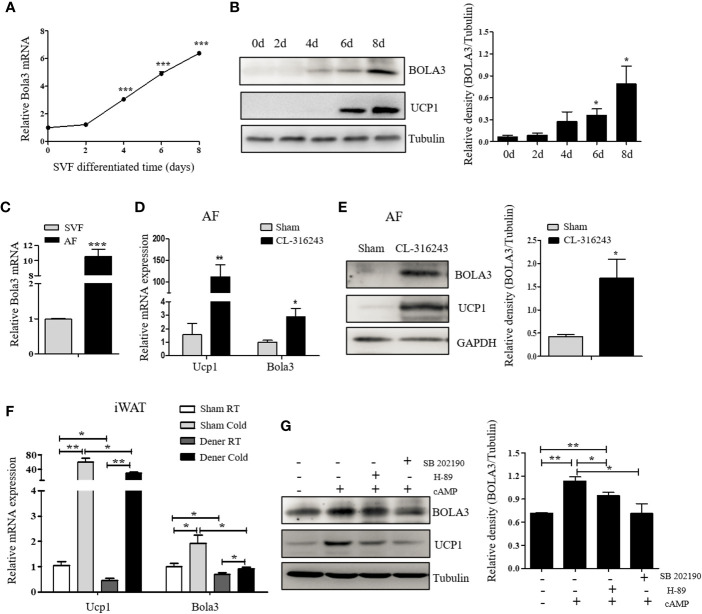
The expression and modulation of Bola3 in thermogenic adipocytes. **(A, B)** SVF cells from inguinal WAT (iWAT) were induced to differentiate into beige adipocytes. Relative Bola3 mRNA (n=3) and protein levels during the time courses of beige adipocyte differentiation. **(C)** Relative Bola3 mRNA expression in SVF and AF isolated from iWAT of 6-week-old male mice (n=3). **(D, E)** Relative Bola3 mRNA (n=4) and protein levels of BOLA3 in AF isolated from iWAT of CL-316243-injected and the sham-operated mice. **(F)** Relative Bola3 and Ucp1 mRNA levels of the denervated and sham iWAT from mice housed at RT and cold environment (n=4). **(G)** Effects of 20 µM H-89 (PKA inhibitor) and 10 µM SB202190 (p38 MAPK inhibitor) on changes of BOLA3 and UCP1 protein levels induced by 0.5 mM dibutyryl-cAMP in differentiated beige adipocytes for 12 h. Data were presented as means ± SEM. *p < 0.05, **p < 0.01, and ***p <0.001. The results are representative of at least three independent repeats in the cell experiments.

Preadipocytes resided in SVFs cells from iWAT have the distinct capacity to differentiate into beige adipocytes (30). We next isolated SVF cells from iWAT of male C57BL/6J mice and differentiated them to examine Bola3 expression. After an 8-day induction with beige lipogenic procedures, we found that Bola3 mRNA expression was continuously increased (**Figure 2A**). Concomitantly, BOLA3 protein levels presented an increasing trend during the time course of beige adipocyte differentiation, which were similar to the induction of thermogenic marker UCP1(**Figure 2B**). We further isolated the SVF cells and mature adipocyte fraction (AF) from iWAT of male C57BL/6J mice, and found that Bola3 mRNA level was nearly 10-fold higher in AF than in SVF, indicating that Bola3 was predominantly expressed in mature adipocytes among all the resident cell types (**Figure 2C**). Furthermore, BOLA3 mRNA and protein levels were both significantly upregulated in AF isolated from iWAT of CL-316243-injected mice, a browning model with active adrenergic signaling (**Figures 2D, E**). These findings further supported the specific role of Bola3 on the browning program of white adipocytes.

To determine whether sympathetic signaling was required for Bola3 induction in thermogenic adipocytes, here we performed denervation studies in one side of iWAT to analyze Bola3 expression under basal and cold-induced state. Compared to the sham side, Bola3 mRNA levels were significantly decreased in the denervated side both at RT and chronic cold environment, which were in consistent with Ucp1, indicating that the sympathetic signaling was necessarily required for Bola3 expression in innervated beige fat (**Figure 2F**). Norepinephrine from sympathetic nerves can activate β**_3_**-adrenergic receptor, increase cyclic adenosine monophosphate (cAMP) concentration, and enhance the activity of cAMP-dependent protein kinase A (PKA) pathway, thereby controlling the expression of thermogenic genes (31). To further investigate the modulation of Bola3 expression in differentiated beige adipocytes, we treated them with cAMP that enhanced the activity of PKA and p38 mitogen-activated protein kinase (p38 MAPK) pathway. Meanwhile, we assessed the effects of PKA antagonist (H-89) and p38 MAPK antagonist (SB202190) on BOLA3 modulation. Results showed that treatment of cAMP promoted a robust induction of BOLA3 protein level in beige adipocytes, while this effect was almost blocked by H-89 and SB202190, in parallel with UCP1, suggesting that Bola3 might be regulated through the cAMP/PKA/p38 MAPK pathway (**Figure 2G**). Overall, these findings might further support the potential role for Bola3 in the browning program as it responds to the adrenergic-induced, cAMP-mediated signaling.

### Bola3 Deficiency Led to Impaired Thermogenesis in Normal Differentiated Beige Adipocytes

**Figure 3:**
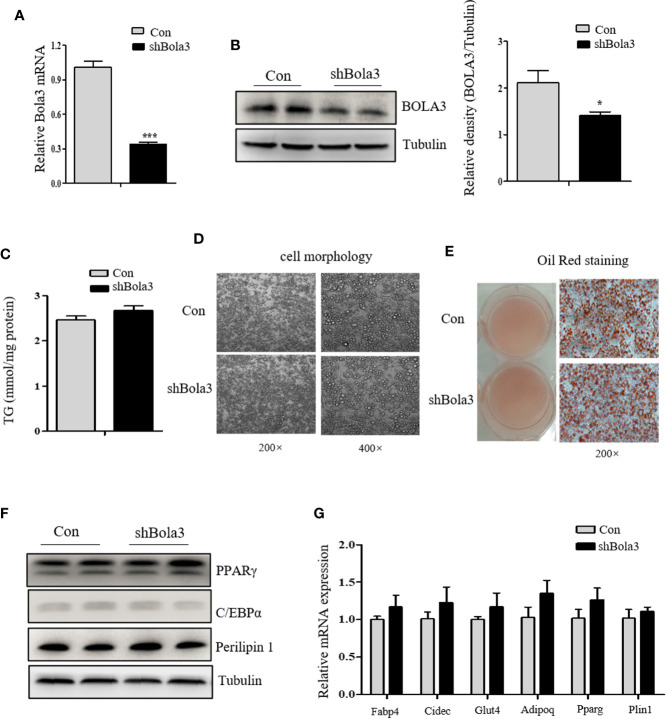
Bola3 knockdown did not affect the lipogenesis process in differentiated beige adipocytes. **(A, B)** Relative Bola3 mRNA (n=4) and protein levels in beige adipocytes differentiated from inguinal WAT (iWAT) stromal vascular fraction (SVF) cells with infection of mouse Bola3 lentiviral shRNA. **(C**–**E)** TG concentration (n=4) and representative images of cell morphology and Oil Red O staining in beige adipocytes with Bola3 knockdown. **(F)** Protein levels of the lipogenic genes (PPARγ, C/EBPα and Perilipin 1) in beige adipocytes infected with mouse lentiviral Bola3 shRNA. **(G)** Quantitative PCR analysis of the lipogenic genes (Fabp4, Cidec, Glut4, Adipoq, Pparg and Plin1) expression in beige adipocytes infected with lentivirus expressing shBola3 or control (n=4). Data were presented as means ± SEM. *p < 0.05 and ***p <0.001. The results are representative of at least three independent repeats in the cell experiments.

**Figure 4:**
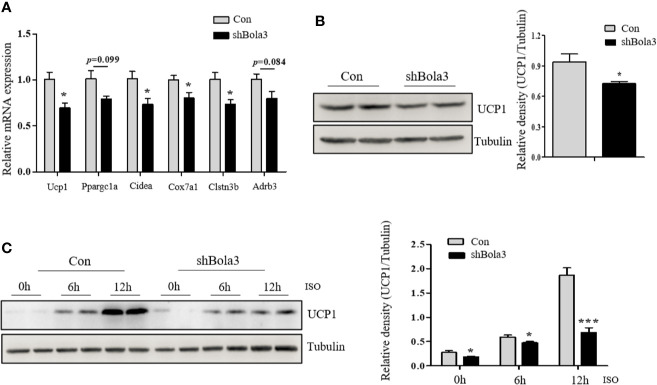
Bola3 was required for maintaining beige adipocyte thermogenesis. **(A)** Quantitative PCR analysis of the thermogenic genes (Ucp1, Ppargc1a, Cidea, Cox7a1, Clstn3b and Adrb3) expression in beige adipocytes with the infection of mouse Bola3 lentiviral shRNA (n=4). **(B)** Western blot and quantification analysis of UCP1 protein levels in beige adipocytes infected with mouse lentiviral shRNA. **(C)** The protein levels of UCP1 were immunoblotted after 5 µM ISO treatment for 6 and 12 h. Data were presented as means ± SEM. *p < 0.05 and ***p <0.001. The results are representative of at least three independent repeats in the cell experiments.

To reveal the role for Bola3 in WAT browning program, we performed *in vitro* loss of function experiments. Firstly, we efficiently knocked down Bola3 expression in SVF cells from iWAT with lentiviral-delivered shRNA and differentiated them into mature beige adipocytes (**Figures 3A, B**). Bola3 reduction did not affect lipid accumulation as determined by triglyceride quantification, cell morphology and Red Oil O staining (**Figures 3C–E**). Western blot analysis showed that the protein levels of adipocyte differentiation related genes such as PPARγ, C/EBPα, and Perilipin 1 remained unchanged with Bola3 knockdown (**Figure 3F**). In accordance with this, there were no significant difference of mRNA levels of lipogenesis related genes (Fabp4, Cidec, Glut4, Adipoq, Pparg, and Plin1) between the groups (**Figure 3G**). However, mRNA expression of thermogenic genes (Ucp1, Cidea, Cox7a1, and Clstn3b) were downregulated to a certain extend due to Bola3 deficiency (**Figure 4A**). UCP1 protein expression was likewise downregulated by Bola3 knockdown (**Figure 4B**). Furthermore, we observed that UCP1 protein levels in Bola3-deficient beige adipocytes were robustly inhibited in the presence of ISO, which activate thermogenesis *via* β**_3_**-adrenergic signaling (**Figure 4C**). These results together suggest that Bola3 knockdown exhibits impaired thermogenic gene program while remains normal lipogenesis capacity in differentiated beige adipocytes.

### Bola3 Mediated Browning Program by Regulating Mitochondrial Homeostasis and Lipolysis

**Figure 5:**
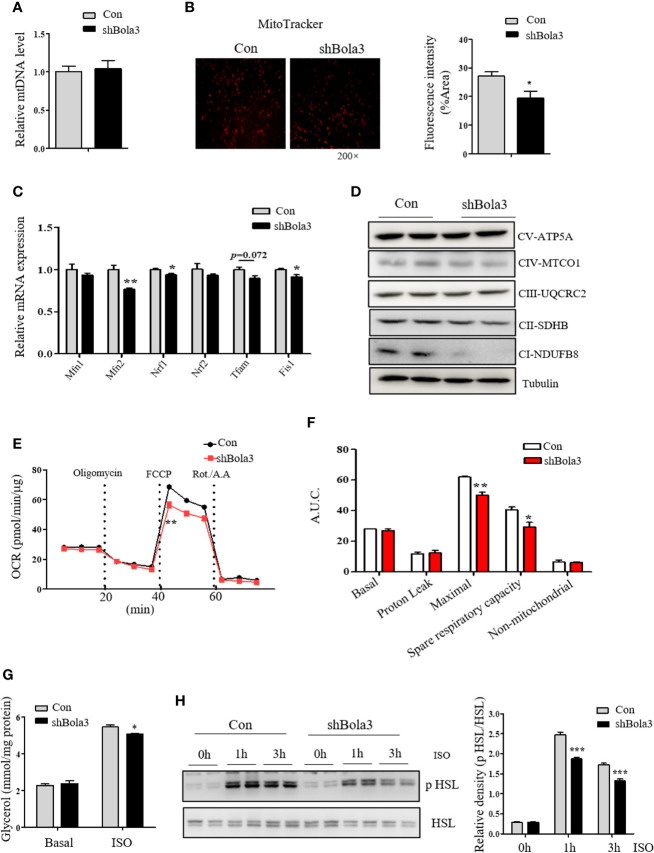
Bola3 regulated thermogenic programming by maintaining mitochondrial homeostasis. **(A, B)** Mitochondrial DNA (mtDNA) levels (n=4) and representative fluorescent images, fluorescence intensity of MitoTracker staining in beige adipocytes differentiated from iWAT SVF cells infected with or without Bola3 lentiviral shRNA. **(C, D)** Quantitative PCR analysis of mitochondrial genes expression (n=3) and protein levels of mitochondrial OXPHOS complexes in beige adipocytes with Bola3 knockdown. **(E, F)** OCR measurement in Bola3-deficient beige adipocytes under 4-day differentiation. **(G, H)** Glycerol release (n=4) and p HSL 563, HSL protein levels in differentiated beige adipocytes infected with Bola3 lentiviral shRNA or control under basal and ISO-stimulated state. Data were presented as means ± SEM. *p < 0.05, **p < 0.01 and ***p <0.001. The results are representative of at least three independent repeats in the cell experiments.

As shown above, we found a suppression of thermogenic gene program in Bola3-deficient beige adipocytes. Next, we explored the possible insights underlying Bola3-mediated effects on thermogenic programming. Beige adipocyte is characterized by a sufficient number of functional mitochondria, which are essential for adaptive thermogenesis (32). As Bola3 was identified as a key regulator of Fe-S-specific mitochondrial respiratory chain, we investigated mitochondrial content and activity in Bola3-deficient beige adipocytes. Although mtDNA content had no difference between shBola3 infection and control group (**Figure 5A**), diminished fluorescence intensity *via* MitoTracker red-staining was observed in Bola3-deficient adipocytes, suggesting attenuated mitochondrial formation in beige adipocytes with Bola3 deletion (**Figure 5B**). Furthermore, quantitative PCR analysis showed that mitochondria related genes such as Mfn2, Nrf1, and Fis1, were inhibited by Bola3 reduction (**Figure 5C**). Consistent with the gene expression data, the protein levels of representative Complex I subunit, NDUFB8, and Complex II subunit, SDHB, were significantly decreased in Bola3-deficient beige adipocytes (**Figure 5D**), which was consistent with previous conclusion in other type of cells (18). In a mitochondrial stress test, basal respiration of beige adipocytes with Bola3 ablation was not altered, however Bola3 deficiency inhibited the ability to respond to FCCP, as demonstrated by the reduced maximal respiration, as well as the diminished spare respiratory capacity (**Figures 5E, F**). Overall, these data suggest that Bola3 plays an indispensable role in maintaining mitochondrial homeostasis, and thereby regulates beige adipocyte thermogenesis.

In addition to the effect of Bola3 knockdown on mitochondrial activity, we further explored its role in regulating the activity of lipolysis under basal and ISO-induced state. Activation of adrenergic signaling has been known to promote lipolysis *via* PKA-mediated phosphorylation of HSL (33). In beige or brown adipocytes, free fatty acids (FFAs) released by lipolysis can activate existing UCP1 and drive heat production in mitochondria (22). Results showed that there was no difference in glycerol concentration under basal state, while Bola3 deficiency repressed ISO-induced glycerol release to a certain degree (**Figure 5G**). We next investigated the effect of Bola3 deletion on downstream intracellular signaling activation. It was found that ablation of Bola3 led to inhibited responses to adrenergic stimulation in beige adipocytes, as demonstrated by the decreased levels of HSL phosphorylation at S563 (**Figure 5H**). Hence, we demonstrated that Bola3 deficiency in beige adipocytes impairs isoproterenol-stimulated lipolysis, implying this might be another aspect by which Bola3 regulates beige adipocyte thermogenesis.

### BOLA3 mRNA Levels in Human Adipose Depots Were Positively Correlated With Thermogenic Gene Program

**Figure 6:**
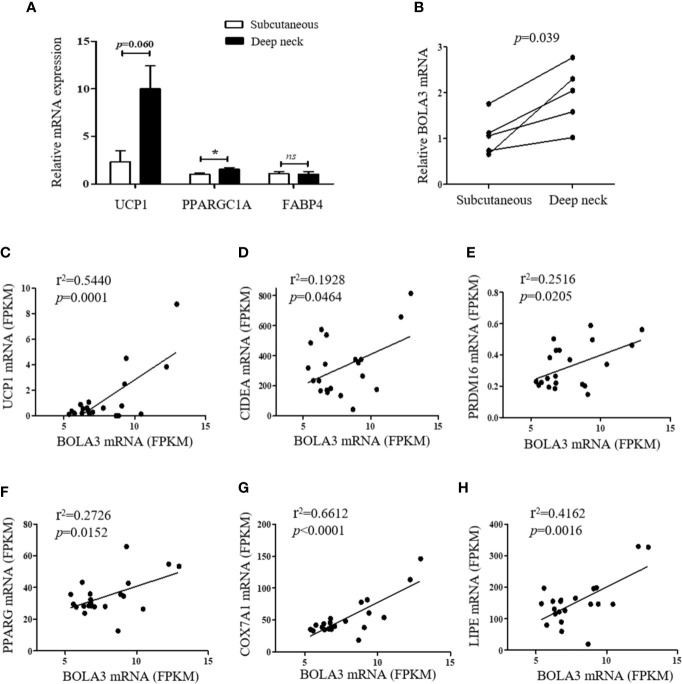
BOLA3 mRNA levels were positively associated with thermogenic gene program in human adipose depots. **(A)** Quantitative PCR analysis of thermogenesis related genes (UCP1 and PPARGC1A) and lipogenesis related gene (FABP4) in human deep neck fat and the paired subcutaneous fat (n=5). **(B)** Gene expression analysis showed higher BOLA3 expression in human deep neck fat compared to the paired subcutaneous fat (*p*=0.039, n=5). Data were presented as means ± SEM. *p < 0.05, ns, not significant. **(C–G)** Relative BOLA3 expression is plotted against the expression of thermogenesis related genes (UCP1, CIDEA, PRDM16, PPARG, and COX7A1). **(H)** Relative BOLA3 expression is plotted against the expression of gene involved in lipolysis (LIPE, namely HSL). Gene expression levels are represented as fragments per kilobase of exon per million mapped reads (FPKM) value from RNA-seq; Linear regression analysis was performed; r^2^ and *p* values were presented in the figure (n=21 omental fat samples from lean and obese human subjects).

Finally, we investigated whether the role for Bola3 in browning program that we observed in mice might occur in human subjects. We simultaneously obtained the subcutaneous and deep neck adipose depots from patients who underwent thyroidectomy. The subcutaneous fat and deep neck fat were regarded as white adipose and brown adipose respectively (27). RT-qPCR analysis confirmed that the thermogenesis marker genes, UCP1 and PPARGC1A, had higher transcript levels in deep neck fat compared to the paired subcutaneous fat, while the adipocyte differentiation marker FABP4, had no difference between groups (**Figure 6A**). We observed that BOLA3 mRNA levels were higher in human deep neck fat than in the paired subcutaneous fat, which demonstrated that higher BOLA3 levels were correlated with greater tissue oxidative capacity in human adipose tissue samples as well (**Figure 6B**). Furthermore, we investigated whether BOLA3 mRNA levels were correlated with genes which were involved in the process of thermogenesis and lipolysis in omental adipose depots from lean and obese human subjects. Association analysis showed that BOLA3 mRNA levels were positively correlated with the expression of thermogenesis related genes (UCP1, CIDEA, PRDM16, PPARG, and COX7A1) and lipolysis related gene LIPE, namely HSL (**Figures 6C–H**). Thus, we concluded that BOLA3 might play a regulatory role in thermogenic programming in human adipose depots as well.

## Discussion

In this study, we found that Bola3 expression was significantly induced in iWAT upon thermogenic activation, while Bola3 deficiency *in vitro* led to impaired thermogenesis activity of beige adipocytes. We further identified the positive correlation between BOLA3 expression and thermogenic gene program in human adipose depots. Taken together, we demonstrate that Bola3 plays a regulatory role in the thermogenic program of white fat in both mice and human subjects.

Beige fat has been regarded as a promising intervention target for obesity (34), because it is highly inducible under multiple stimuli, in the process, mitochondrial biogenesis is the key to beige adipocyte development. As mitochondria are involved in a complex communication network, continuously communicating and interacting with other organelles, even distinct tissues through multiple ways (35), hence the maintenance of mitochondrial homeostasis deserves to be further investigated in order to reveal the underlying pathologic mechanism and potential therapeutic interventions. The mitochondrial Fe-S cluster and its targeted Fe-S proteins are involved in a wide range of metabolic activities (36). The mitochondrial Fe-S cluster assembly machinery consists of about 17 proteins that operate in complex steps for the maturation process of Fe-S targeted proteins (16), while studies on the role of these genes in adipocyte metabolism are still lacking. Among 17 genes, Bola3 expression was most significantly induced in iWAT upon browning inputs. However, few studies have focused on Bola3 function, other than several case reports of multiple mitochondrial dysfunction syndrome due to BOLA3 gene mutation (19). Recently, the first mechanism study demonstrated that Bola3 acted as a crucial lynchpin in the process of endothelial metabolic re-programming in pulmonary hypertension (21). Hence, the dramatic induction of Bola3 expression in WAT browning program, clues that it may be involved in adipose tissue re-programming under thermogenic activation.

This observation promotes us to detect the modulation of Bola3 expression in thermogenic adipocytes. In response to cold exposure, the hormone norepinephrine released from sympathetic nerve system primarily acts on β**_3_**-adrenergic receptor, leading to the activation of PKA and p38 MAPK signaling pathway. This adrenergic signaling is able to activate many thermogenesis related genes (31). We observed that Bola3 was predominantly enriched in mature adipocyte fraction, and thermogenic activation could further enhance its expression in this fraction. Furthermore, we found that Bola3 levels were diminished after iWAT denervation, likewise, block of PKA and p38 MAPK signaling pathway blunted cAMP-mediated induction of BOLA3 expression in differentiated beige adipocytes, which were similar to the pattern of UCP1 expression. Thus, Bola3 could be identified as an adrenergic signaling-targeted gene, which further indicates it may play a significant role in beige adipocyte development.

Next, our findings extend our understanding of the role for Bola3 in beige adipocyte development. It has been reported that BOLA3 mutant fibroblasts show ~30% (Complex I), 50% (complex II), and ~60% (complex III) of normal mitochondrial enzyme activity (18). Major functions of mitochondria in beige adipocytes include differentiation, fatty acid oxidation and thermogenesis (6). Our *in vitro* study demonstrated that Bola3 knockdown by lentivirus expressing shRNA impaired the thermogenic program by the following evidences: 1) Reduced expression of thermogenic genes, as represented by Ucp1, Cidea, Cox7a1, and Clstn3b, a new identified marker gene (37); 2) Disrupted mitochondrial homeostasis, including the decreased levels of mitochondrial components and impaired oxidative metabolism; 3) Inhibited responses to isoproterenol stimulation, in term of lipolysis and FFAs-driven thermogenesis. Collectively, these results suggest that Bola3 plays an indispensable role in regulation of beige adipocyte thermogenesis, possibly by maintaining mitochondrial homeostasis and isoproterenol-mediated lipolysis.

In addition to mice study, we expect to verify our findings in human adipose tissue samples as well. Image-based studies with positron emission tomography-computed tomography (PET-CT) reveals that UCP1-expressing brown-like adipose depots are distributed in the supraspinal, pericardial, and neck regions of adult humans (38, 39). White adipose depots from human and mice have opposing patterns of “browning” gene program (40), showing that human omental fat displays a beige signature with higher expression of thermogenic genes (UCP1, PPARGC1A, TFAM, and TBX1) than the paired subcutaneous fat (41). Our results demonstrated that Bola3 mRNA levels were higher in human deep neck brown fat than in subcutaneous white fat, and they were strongly correlated with thermogenesis related genes (UCP1, CIDEA, PRDM16, PPARG, COX7A1, and LIPE) levels in the omental adipose depots from lean and obese human subjects. These results reinforce that BOLA3 may have a positive effect on regulating tissue oxidative capacity in human subjects as well.

Recently there is another independent study has demonstrated BOLA3 pathway-mediated mitochondrial lipoylation contributes to age-associated decline in brown adipose thermogenesis. As it mentions, there are no significant difference in Ucp1 transcript levels in BAT between the young and old mice (42). However, another study has shown that BAT from the aged mice has decreased Ucp1 mRNA levels (43). Therefore, the expression patterns of thermogenic gene program in BAT from the aged mice, remain to be further clarified. In our study, we found that Bola3 plays an indispensable role in WAT browning program. Obviously, there are two points that should be regarded as the novelty of our study. Firstly, although brown and beige fat are major tissues of adaptive thermogenesis, they also have distinguishing phenotypes and functional features. Brown adipocytes function well under basal conditions, while beige adipocytes express thermogenic components only under stimulation (32). Genetic studies show that genetic variation between mouse strains influence UCP1 levels in WAT but not in BAT under cold exposure, suggesting the different developmental and adaptive mechanism (44). Secondly, aging is a progressive and multifactorial process. In adipose tissue, mitochondrial dysfunction is one of the most highly investigated aging factors (42, 45), however, there exists some other factors as well. For instance, it was recently reported that adipocyte progenitors undergo an age-associated, senescence-like phenotype for age-dependent browning failure (46). Therefore, the pathogenetic mechanisms of age-associated reduction in brown fat thermogenesis will be much more complicated, or there may be different BOLA3 pathway-mediated mechanism involved in WAT browning program in our study.

However, there are some limitations to consider. First, the reason why Bola3 level fluctuation has such a great impact on mitochondrial activity remains unknown and there are several functional marks under thermogenic stimuli that we did not assess, such as mitochondrial morphology and oxygen consumption rate in Bola3-deficient beige adipocytes. The mechanistic roles of Bola3 in beige adipocyte-specific mitochondrial function need to be further explored. Second, our data from human adipose samples are limited to mRNA levels of BOLA3 and thermogenic genes. The correlation analysis between BOLA3 expression and clinical metabolic assessment will be strengthened within these or more human adipose samples. We hope future studies will provide a better understanding of Bola3’s role on beige adipocyte metabolism.

## Data Availability Statement

The original contributions presented in the study are included in the article. Further inquiries can be directed to the corresponding authors.

## Ethics Statement

The studies involving human participants were reviewed and approved by Human Research Ethics Committee of Shanghai Jiao Tong University Affiliated Sixth People’s Hospital and Shanghai Seventh People’s Hospital. The patients/participants provided their written informed consent to participate in this study. The animal study was reviewed and approved by Animal Care Committee of Shanghai Jiao Tong University Affiliated Sixth People’s Hospital.

## Author Contributions

NB performed the experiments, analyzed the results, and wrote the manuscript. JM performed the experiments and analyzed the data. MA and JX assisted the RNA-seq data analysis. FH, YX, and SC assisted the experiments. QL, XL, JH, and WJ contributed the clinical samples. YB reviewed and edited the manuscript. YY conceived the study, wrote, and revised the manuscript. All authors contributed to the article and approved the submitted version.

## Funding

This study was supported by grants from the National Natural Science Foundation of China (No. 82070896, No. 81974122, No. 81670778, No. 81603476).

## Conflict of Interest

The authors declare that the research was conducted in the absence of any commercial or financial relationships that could be construed as a potential conflict of interest.
